# Melatonin: A Myriad of Functions to Discover

**DOI:** 10.3390/antiox13030360

**Published:** 2024-03-18

**Authors:** Adrián Santos-Ledo, Marina García-Macia

**Affiliations:** 1 Instituto de Neurociencias de Castilla y León (INCyL), 37007 Salamanca, Spain; 2 Department of Human Anatomy and Histology, Universidad de Salamanca, 37007 Salamanca, Spain; 3 Institute of Functional Biology and Genomics (IBFG), Universidad de Salamanca-CSIC, 37007 Salamanca, Spain; 4 Department of Biochemistry and Molecular Biology, Universidad de Salamanca, 37007 Salamanca, Spain; 5 Centre for Biomedical Investigations Network on Frailty and Ageing (CIBERFES), 28029 Madrid, Spain

Melatonin is an indoleamine that has captured our attention since 1958 [1]. It was discovered as one of the main regulators of circadian rhythms. At that time, it was simply defined as the sleep–wake regulator hormone, as melatonin is mainly secreted by the pineal gland during the night. Its role in seasonal reproductive functions was also found very soon after. Later, the extra-pineal synthesis of melatonin was discovered (immune system, retina, etc.), together with its production in other organisms, such as bacteria and plants. This fact changed the idea that melatonin was just a hormone that synchronized processes related with the circadian and seasonal rhythms, and brought up the role of melatonin as a very potent antioxidant [2]. The use of oxygen by organisms entails an important cost due to the consequent production of free radicals. To avoid or reduce the detrimental effects of oxidative stress, cells developed specific antioxidant systems, for instance, enzymes such as superoxide dismutase or catalase and, of course, melatonin. This hormone probably appeared in photosynthetic bacteria to prevent oxygen toxicity around 3 billion years ago [3]. Thus, melatonin’s first and more primitive function is protecting cells against toxic products, and it was supplemented with a variety of other roles during evolution.

Melatonin’s main role as an antioxidant is the focus of many pathological studies, such as in metabolic, degenerative, and cardiovascular disorders, as well as in cancer, where there is impaired redox homeostasis. Melatonin’s antioxidant capacity is more effective than that of vitamin E, and it shows intracellular and extracellular activity. It can scavenge ROS directly due to its indole ring, and it can also stimulate the expression of antioxidant enzymes. Furthermore, melatonin can improve mitochondrial homeostasis, which is the main source of free radicals. Despite melatonin’s potential as a therapeutic approach for many diseases linked to increased oxidative stress, more studies are required to establish the putative clinical application of this indoleamine. For this reason and to understand the role of melatonin as an antioxidant, we set up this Special Issue.

We have encountered a myriad of articles about the different uses of melatonin as a therapeutic approach in metabolic diseases, cancer, and neurological afflictions. Some authors explored the conserved role of melatonin as an ROS scavenger against sulfur- and nitrogen-mustard-induced toxicity (Contribution 1) and found that melatonin could be used as a medical countermeasure for blister agent poisoning. In line with the antioxidant functions, one of our articles describes, for the first time, the presence of melatonin synthesis in *Archaea* (Contribution 2), which not only informs us about the primitive origin of the molecule, but also about its conservation and robust role as an antioxidant.

Obesity was an interesting target for our authors’ studies, particularly the prevention of this medical condition through stimulating thermogenesis (Contribution 3). This implies the use of fat through the mitochondria to produce heat instead of energy (a futile cycle). The use of melatonin as a molecule to stimulate thermogenesis can be a suitable treatment for obesity. Our authors showed how melatonin can stimulate these processes to enhance mitochondrial fusion, but in a dose-dependent manner.

Two biomedical fields very interested in melatonin as possible therapy are neuroscience and cancer. Interestingly, the indoleamine seems to have opposing functions, antioxidant and pro-oxidant, depending on the type of studied cell. In this editorial, we provide a thorough review about sepsis-associated encephalopathy, which is a life-threating disfunction caused by infection (Contribution 4). Melatonin was used as a therapeutic substance in many studies, but most of the information came from preclinical studies with animals. Thus, the authors claim the necessity to implement more studies in humans. The manuscripts in our Special Issue differ in the melatonin capabilities in contexts that have common aspects: neurodevelopment and the regeneration of the nervous system. Establishing circadian rhythms is crucial for neurodevelopment. For instance, melatonin enhances important clock proteins also known for their antioxidant capabilities. In particular, individuals with neurodevelopmental disorders such as autism spectrum disorder (ASD), schizophrenia, and bipolar disorder showed lower levels of melatonin secretion and disrupted circadian rhythms (Contribution 5).

Melatonin was used to decipher the relevance of free radicals during the regeneration of optic nerves (Contribution 6). Reactive oxygen species are usually associated with cellular damage, but, currently, they are acquiring more functional roles. When the optic nerve becomes crushed, the regeneration process starts and oligodendrocytes need to succumb and differentiate from new OPCs. Then, they can properly re-myelinate the optic nerve. When melatonin reduces oxidative stress, the regenerative process becomes impaired because damaged oligodendrocytes remain alive.

Melatonin’s antioxidant properties have been explored in many studies. However, there are few clues about the pro-oxidant functions of melatonin functioning as an oncostatic. These are recapitulated in the review we present in our Special Issue (Contribution 7). These mechanisms include different pathways that act through melatonin receptors, as well as sirtuins and the anti-Warburg effect. Besides this, the synergy between melatonin and other antitumoral treatments is explored in very aggressive cancers such as triple-negative breast cancer (TNBC) (Contribution 8). New melatonin derivates such as agomelatine, traditionally used as an antidepressant, are being tested as alternative tumoral treatments (Contribution 9). Agomelatine was tested in in vitro colorectal cancer models, and it was able to reduce the proliferation through NF-Kb inhibition. Thus, these new molecules show a double function, antidepressant and antitumoral, which may be due to the modification of the tumor environment or the alteration of the immune response.

This last function is also tackled in our Special Issue with a different approach. The optimization of bird production has deep economic repercussions, as chickens are one of the most consumed meats worldwide. Birds are very sensitive to light and their immune system is very susceptible to different wavelengths, especially bursal B-lymphocytes, which can undergo apoptosis depending on the type of light. This manuscript showed how chickens exposed to a determined light had an increased melatonin level, which reduced B-lymphocyte oxidative stress and prevented apoptosis (Contribution 10).

In summary, with this Special Issue, we try to explore the most recent roles of melatonin as an antioxidant, and we provide a great deal of new information: from melatonin synthesis in a new domain to recent uses of melatonin as a therapy and synergistic treatment for cancer. Thus, we are sure that this indoleamine will be in the spotlight for much more time to come.
